# Making a Difference from Day One: The Urgent Need for Universal Neonatal Hearing Screening

**DOI:** 10.3390/children11121479

**Published:** 2024-12-03

**Authors:** Michail Athanasopoulos, Pinelopi Samara, Georgios Batsaouras, Ioannis Athanasopoulos

**Affiliations:** 1 Department of Audiology, Otology, Neurotology & Cochlear Implant Unit, Athens Pediatric Center, 15125 Athens, Greece; miathanasopoulos@gmail.com (M.A.); dr.iathanasopoulos@gmail.com (I.A.); 2 Children’s Oncology Unit “Marianna V. Vardinoyannis-ELPIDA”, Aghia Sophia Children’s Hospital, 11527 Athens, Greece; 3 Department of Otolaryngology, University Hospital of Patras, 26504 Patras, Greece; batsaouras_georgios@yahoo.gr

**Keywords:** neonatal hearing screening, early detection, congenital hearing loss, early intervention, transient evoked otoacoustic emissions, automated auditory brainstem response, hearing aids, cochlear implants

## Abstract

Neonatal hearing screening (NHS) is a critical public health measure for early identification of hearing loss, ensuring timely access to interventions that can dramatically improve a child’s language development, cognitive abilities, and social inclusion. Beyond clinical benefits, NHS provides long-term advantages in education and quality of life. Given that congenital hearing loss affects approximately 1–2 in every 1000 newborns worldwide, the case for universal screening is clear. Countries like the United States and Australia have successfully implemented NHS, leading to earlier diagnoses, improved language development, and better educational outcomes. In Europe, while many nations have adopted NHS, consistency remains a challenge. Countries such as Norway and the United Kingdom stand out for their well-established systems, whereas others, like Greece, have made notable progress but have yet to mandate NHS nationwide. This highlights the need for cohesive national policies across Europe to ensure universal coverage. Screening methods such as Transient Evoked Otoacoustic Emissions (TEOAE) and Automated Auditory Brainstem Response (AABR) are established tools for detecting hearing impairments in neonates. Despite their demonstrated efficacy, NHS remains inconsistent globally, particularly in low- and middle-income regions that lack mandatory policies or access to reliable screening technologies. This perspective advocates for the urgent need to make NHS mandatory in all countries, emphasizing its societal benefits and cost-effectiveness. Early diagnosis supports prompt intervention, like hearing aids or cochlear implants, which are most effective when implemented before six months. It also empowers families to make informed decisions, fosters educational inclusion, and mitigates the social and emotional challenges of undiagnosed hearing loss. Policymakers, healthcare providers, and international organizations must prioritize universal NHS to ensure no child is left behind due to unaddressed hearing loss.

## 1. Introduction

Neonatal hearing screening (NHS) is an essential public health initiative designed to identify hearing impairments in newborns, allowing for timely intervention that can transform a child’s life. Early detection enables children with hearing impairments to receive critical support for language, cognitive, and social skill development [1]. Despite congenital hearing loss affecting approximately 1–2 in every 1000 newborns worldwide—making it more prevalent than many other screened conditions—NHS is still not universally included in mandatory newborn screening programs [2]. This contrasts with conditions like phenylketonuria (PKU), congenital hypothyroidism, galactosemia, and G6PD deficiency, which, though generally less common, are compulsory for screening to allow for timely intervention [3]. In fact, PKU affects about 1 in 10,000–15,000 newborns [4], and congenital hypothyroidism impacts roughly 1 in 3000–4000 [5], yet both are standard in many screening programs. G6PD deficiency, while variable in prevalence due to genetic factors, is also commonly screened in regions where it is more widespread [6].

The World Health Organization (WHO) estimates that over 34 million children globally live with disabling hearing loss, and that nearly 60% of childhood hearing loss cases could have been prevented through public health measures such as immunization and improved prenatal and neonatal care [7]. In high-income countries, NHS programs have successfully reduced the long-term impacts of hearing loss by ensuring children receive access to hearing aids, cochlear implants, and early speech therapy [8]. However, NHS programs are not consistently implemented worldwide, often due to economic disparities, inadequate policies, or lack of mandatory screening requirements. Expanding access to NHS worldwide could help bridge this gap, ensuring early support and intervention for children with hearing loss across all regions [9].

This essay advocates for NHS to be universally mandated as both an ethical and social imperative, promoting equal opportunities for all children. It emphasizes the importance of accessible and well-coordinated programs, alongside increased public awareness, ensuring that parents understand the value of early screening and are encouraged to utilize these services for their children’s development. Furthermore, this essay highlights strategies to improve NHS accessibility, particularly in countries like Greece, where NHS programs are still evolving.

## 2. The Right to Hear: Advocating for Universal NHS

Mandating NHS worldwide is an ethical obligation. Every child deserves access to healthcare that maximizes their developmental potential, and hearing is fundamental to this growth. The WHO has underscored hearing health as essential to a child’s social inclusion, education, and overall well-being [7]. Early identification of hearing impairment followed by swift intervention offers children an equal start, ensuring they can develop language skills, cognitive abilities, and social interactions alongside their hearing peers. Health equity further supports this call for universal NHS. Currently, NHS availability often depends on a country’s economic resources and healthcare infrastructure, leaving children in low-income regions at a disadvantage. Mandating NHS globally not only aligns with health equity but also asserts the ethical right of every child to access healthcare that promotes their full potential, regardless of socioeconomic or geographic barriers.

By “mandatory”, we refer to establishing a universally accessible, centrally organized NHS system, supported by comprehensive education for families on the importance of early hearing detection. This is not about legal enforcement but rather about creating a framework that encourages informed parental participation and ensures equitable access to early intervention for all children.

## 3. The Comprehensive Benefits of Early Intervention in Child Development

Early intervention following NHS has a transformative impact on every aspect of a child’s development. When hearing impairments are detected within the first few months of life, immediate intervention can begin, offering critical support during a period when the brain is especially receptive to language and developmental input. Research indicates that children with hearing loss who receive intervention by six months of age often achieve developmental outcomes comparable to their hearing peers across various domains [10].

### 3.1. Language and Cognitive Development

Hearing is the “gateway” to language. Early intervention through hearing aids, cochlear implants, and speech therapy enables children to access sound, setting the stage for developing language skills essential for communication and thought. Without intervention, children with hearing impairments face significant barriers to language acquisition, which can impact learning and social interaction [11,12]. When intervention is timely, children can build vocabulary, understand syntax, and develop expressive language [13], establishing a cognitive foundation for lifelong learning and effective problem solving.

### 3.2. Emotional and Psychological Well-Being

Timely support helps children avoid the frustration, isolation, and low self-esteem that often accompany undiagnosed hearing loss [14]. By enabling communication, early intervention empowers children to express their emotions, connect with family members, and build friendships, which fosters confidence and resilience. This psychological well-being is fundamental for developing healthy self-esteem and positively engaging with others [15].

### 3.3. Social Development and Inclusion

Social skills are primarily developed through interaction and communication. Without support, children with hearing impairments may struggle to engage with peers, delaying the development of social skills and often leading to isolation. Early intervention equips these children with essential communication skills, enabling them to form friendships, participate in group activities, and feel included in their communities—promoting social harmony and a strong sense of belonging [16].

### 3.4. Educational Potential and Academic Success

Education relies heavily on language and cognition, both of which are affected by hearing loss. Early intervention ensures children develop language skills in time for school, reducing the need for specialized education later and supporting mainstream classroom success. Studies indicate that children with early-supported hearing loss perform better academically, strengthening their foundation for a fulfilling educational journey [17].

## 4. Ensuring Effective NHS: The Importance of Comprehensive Screening Methods

To maximize the effectiveness of NHS, it is important to adopt a comprehensive approach that combines both Transient Evoked Otoacoustic Emissions (TEOAE) and Automated Auditory Brainstem Response (AABR) tests [18]. This dual-method approach increases the sensitivity of screening, capturing potential cases of hearing impairment that a single test might miss [19,20]. TEOAE tests measure the response of the inner ear (cochlea) to sound stimuli, helping to detect hearing loss. However, TEOAE screening alone may miss cases of auditory neuropathy spectrum disorder (ANSD), a type of neural hearing loss where the cochlea functions properly, but sound signals are not transmitted effectively along the auditory nerve to the brain [21]. AABR tests assess the auditory nerve pathways, which are essential for detecting ANSD and other neural hearing disorders that might otherwise go undetected if only TEOAE are performed. By evaluating both the cochlea (via TEOAE) and the auditory nerve pathways (via AABR), this dual-screening approach provides valuable insights into a child’s overall auditory health [20].

Ideally, NHS should be conducted before a newborn reaches one month of age, with the optimal window starting from the second day of life to ensure early detection and timely follow-up. If the newborn fails the screening (non-pass/refer result), it is recommended to repeat the tests within ten days. Some potential reasons for inconclusive results in NHS could include the presence of residual birth fluids in the ear canal, excessive noise during testing, or the infant’s unsettled state, such as crying or movement. The small size and unique anatomy of newborn ear canals can affect the accuracy of hearing tests, as ear canal collapse may hinder sound [22]. If the follow-up tests indicate a refer result as well, the child should be referred to specialized pediatric audiology centers for further evaluation and intervention. Confirmatory diagnostic testing should be completed by three months of age to enable appropriate intervention by six months [23]. In 2019, the Joint Committee on Infant Hearing recommended a shift from the traditional 1-3-6 screening protocol to a 1-2-3 model, emphasizing earlier intervention and faster follow-up. Programs following the 1-3-6 timeline were encouraged to consider adopting this more expedited approach [24].

If infants pass the initial hearing screening for both ears, it should be communicated that the results suggest normal hearing at the time of testing, and no further assessment is required. However, it is important to inform caregivers that some hearing impairments may develop later in infancy or early childhood. Therefore, parents should remain attentive to their child’s responses to sound and seek professional advice if they have concerns about the infant’s hearing, speech, or language development. If no concerns arise during infancy, parents should consider repeating the hearing tests during the preschool years [19]. For high-risk infants—such as those with a family history of hearing loss, neonatal intensive care unit (NICU) stays, low birth weight, prematurity, increased bilirubin levels, perinatal hypoxia, infections [e.g., meningitis, cytomegalovirus (CMV), and TORCH infections such as toxoplasmosis, rubella, syphilis, varicella-zoster, and herpes simplex], craniofacial and neurodevelopmental anomalies, exposure to ototoxic medications, mechanical ventilation, ECMO (extracorporeal membrane oxygenation), trauma during delivery, severe intracranial hemorrhage, or neonatal convulsions—both initial and more frequent follow-up screenings are essential to promptly detect any late-onset or progressive hearing loss that may not be evident at birth [25].

**Figure 1:**
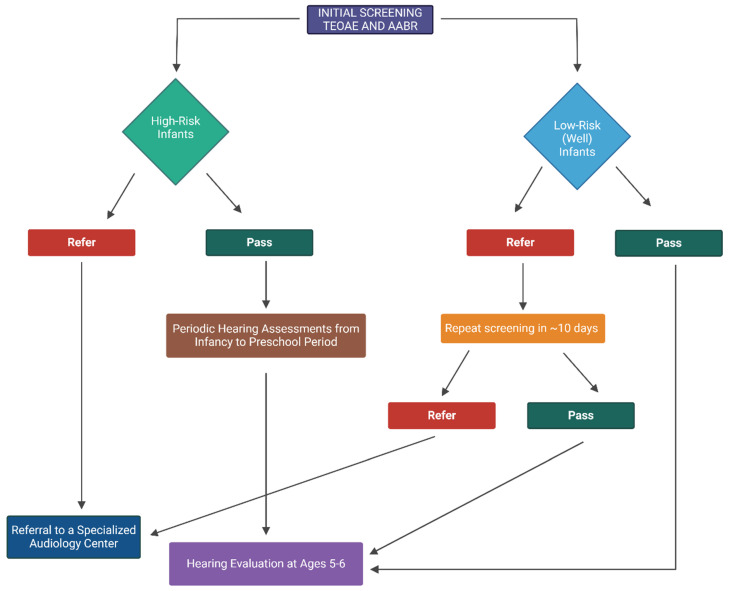
Neonatal hearing screening algorithm for high-risk and low-risk (well) infants. This flowchart outlines the screening process for newborns, distinguishing between high-risk and low-risk groups using both Transient Evoked Otoacoustic Emissions (TEOAE) and Automated Auditory Brainstem Response (AABR) tests. It includes initial screening methods, follow-up protocols, and referral criteria based on the results. Low-risk (well) infants undergo routine screening, while high-risk infants may require more frequent monitoring or diagnostic testing. The algorithm emphasizes early identification, timely intervention, and continuous monitoring through preschool and school-age years for ongoing hearing assessments and support (Created with BioRender.com).

Based on the aforementioned evidence and guidelines, our team applies and proposes the neonatal hearing protocol, as outlined in Figure 1, to optimize early detection and intervention for hearing impairments in both high-risk and low-risk (well) infants. A combined approach using both TEOAE and AABR testing from the beginning is more effective than relying on a single test. This strategy minimizes false referrals, reduces unnecessary retesting, and alleviates the psychological, logistical, and economic burden on families, ultimately ensuring more reliable and early detection.

## 5. The NHS Landscape in Greece: Progress, Current Gaps, and Opportunities for Universal Implementation

NHS policies across Europe vary significantly [26]. The United Kingdom has a well-established NHS program that ensures screening for all newborns. Countries like Norway, Hungary, and the Czech Republic, along with the United States and Australia, have successfully implemented nationwide universal NHS programs, leading to substantial benefits in early detection and intervention (Table 1). While countries in Asia, such as Japan, China, and South Korea, have made progress with nationwide, universal programs, NHS remains limited in many regions (Table 2) [27,28,29,30,31]. By contrast, Greece is still working toward nationwide universal NHS adoption, facing challenges that impede its implementation and underscoring the need for stronger policies and infrastructure to ensure all children have access to early screening [32].

The information presented in the tables was derived from the EUSCREEN Study, a European Union-funded project assessing child vision and hearing screening programs across Europe. Data collection occurred primarily through the EUSCREEN Questionnaire, accessible from March 2017 to December 2018, with submissions extended to June 2019. Additionally, an updated questionnaire was circulated in September 2021 to verify and revise responses, ensuring that the data reflect recent developments in screening policies and implementation [33,34,35,36,37]. While these efforts aimed to capture the most current information, NHS policies are dynamic and differ between countries, highlighting the importance of periodic updates and ongoing monitoring to address regional differences and evolving practices.

The following outlines strategic recommendations to enhance NHS in Greece. These proposals target key challenges, such as advocating for policy reforms, expanding service access, securing sufficient funding, raising public awareness, and improving data collection systems. The objective is to standardize NHS nationwide, ensure timely screenings, bolster support systems for families, and integrate NHS more seamlessly into Greece’s public health framework.

### 5.1. Advocating for Policy Change and Nationwide Commitment

One of the most important actions Greece can take is to advocate for policy change that prioritizes NHS at the national level. By including NHS as part of Greece’s core public health services and ensuring adequate funding for its implementation, the government can make a long-term commitment to improving the hearing health of all children [19]. Engaging with healthcare professionals, policymakers, and advocacy groups will help garner support for NHS, increasing the likelihood of securing the political and financial commitment necessary for nationwide implementation [38].

### 5.2. Improving Access and Standardization of NHS

Currently, NHS is available in some hospitals in Greece, but the lack of a nationwide mandate leads to delayed diagnoses, missed developmental milestones, and social challenges for many children. This situation presents an opportunity for innovative solutions. A focused policy should mandate that every public and private hospital with a maternity clinic and an NICU implement a free NHS program. Nurses, midwives, and other birth attendants should be trained to conduct screenings [39], ensuring that all newborns receive timely hearing assessments, regardless of region or socioeconomic status. Such a strategy would help standardize NHS across the country, reduce disparities, and facilitate early intervention for all children.

### 5.3. Strengthening Funding, Monitoring, and Support Systems

Greece could strengthen its NHS system by seeking funding and technical support from the European Union [40] and international organizations such as the WHO [19]. These entities could offer grants to help scale up the NHS program. Collaborative efforts could focus on standardizing protocols, training more healthcare professionals, and securing funds for equipment and establishing nationwide screening centers. Funding allocation must be closely monitored to ensure effective use specifically for NHS enhancements. Oversight mechanisms should focus on measurable outcomes such as early detection rates, intervention success, and reduction in disparities. Strengthening financial and monitoring systems will help Greece develop a sustainable and efficient NHS framework.

### 5.4. Addressing Public Awareness and Promoting Early Screening

A significant barrier to universal NHS in Greece is the lack of public awareness. Many parents are unaware of the importance of early hearing screening or may be unfamiliar with the process and its benefits. To address this, pediatricians, obstetricians, and other healthcare providers should actively inform and encourage new parents to prioritize NHS for their newborns. These healthcare professionals should be trained in the significance of NHS and identifying at-risk infants. Pediatricians, in particular, can play a crucial role in advising parents to follow up with hearing screening if it is not automatically provided, ensuring they comprehend the long-term benefits of early detection [41]. In addition, a nationwide public awareness campaign is essential to educate families about the critical role of NHS, the potential consequences of undiagnosed hearing impairments, and the resources available for support. This campaign should utilize multiple media channels—TV, radio, social media, and community outreach—to maximize its influence. Providing clear information on the signs of hearing loss and the NHS process will empower parents to seek screening for their newborns, ensuring early detection and timely intervention.

### 5.5. Data Collection and Specialized Care for Families

Another key element for improving NHS in Greece is the development of a national database, managed centrally by the Ministry of Health and overseen by specialists in the field. This database would track the progress of screening programs, monitor outcomes, and identify gaps in coverage. It would also help identify trends in hearing loss and the success of interventions, facilitating evidence-based adjustments to the program [42]. Such a system would provide valuable insights for ongoing improvement and help ensure equitable access to NHS across all demographics.

After a diagnosis of hearing impairment, families in Greece need accessible resources to help them navigate the next steps. This includes counseling, access to speech therapy, audiological services, support groups, and social workers who can provide guidance on managing the emotional, financial, and social aspects of the diagnosis [43]. Strengthening this support system would not only help parents make informed decisions about their child’s care, but also ease the emotional burden that often accompanies a hearing loss diagnosis. Additionally, the establishment or expansion of specialized pediatric audiology centers in all major peripheral regions of Greece would ensure that families have access to focused care and services close to home. Implementing family-centered care, where parents are empowered with the knowledge and tools they need, could help reduce stress and improve outcomes for both children and families.

## 6. Practical Steps for Effective Early Intervention and Supporting Child Development

Achieving the benefits of early intervention requires a structured, multi-faceted approach. The effectiveness of early intervention for hearing impairments relies on a thorough understanding of the various causes of congenital hearing loss, which may include genetic factors (both syndromic and non-syndromic), viral infections, perinatal complications, prematurity, exposure to ototoxic medications, and maternal health conditions [44]. The prognosis can vary significantly depending on the underlying cause, making early diagnosis and targeted intervention critical for optimizing developmental outcomes. The following components are essential for ensuring effective early intervention [19] and supporting the child’s developmental trajectory.

### 6.1. Immediate Post-Diagnosis Support

After a diagnosis of hearing loss, it is important to provide families with immediate support, including counseling, access to resources, and a personalized action plan tailored to the specific cause of the hearing impairment. If the hearing loss is linked to a genetic mutation [45], cochlear implants or hearing aids may be recommended. A comprehensive evaluation should also be performed to identify potential systemic issues, as many genetic causes of hearing loss can affect other organ systems. For hearing loss caused by intrauterine viral infections, antiviral treatments may be considered. In cases of congenital CMV, regular audiological testing is essential to monitor for delayed-onset hearing loss [46]. Early specialist involvement helps parents make informed decisions about the most appropriate interventions.

### 6.2. Access to Multidisciplinary Care Teams

The best outcomes in early intervention come from a team approach. A multidisciplinary team—comprising pediatric otolaryngologists, audiologists, speech therapists, pediatricians, and mental health professionals—ensures that the child’s care is comprehensive and personalized. This holistic approach addresses not only the child’s hearing and language development but also their social and emotional growth. For example, children with hearing loss resulting from perinatal complications or prematurity may require additional support to address developmental delays. By adopting a team-based approach, each child’s unique needs can be effectively met, ensuring comprehensive care across all areas of development.

### 6.3. Parental and Family Education Programs

A critical component of early intervention is educating parents and families. Family involvement can improve outcomes for children with hearing loss. Education programs should teach parents how to support language and social development at home and how to use alternative communication tools if necessary. Specifically, if a child is diagnosed with hearing loss due to ototoxic medications, parents should be informed about the potential for progressive hearing loss, allowing them to monitor the child closely and assess whether hearing devices remain effective. These programs empower families to create a language-rich environment at home that supports the child’s ongoing development and strengthens the bond between parent and child.

### 6.4. Integration of Technology in Therapy

Technological advances, such as digital hearing aids, cochlear implants, and mobile applications, play a vital role in supporting the development of children with hearing impairments. Additionally, telehealth services, including remote device adjustments, can help bridge the gap for underserved families, providing access to therapy, counseling, and support even in remote areas. Early and effective use of these technologies can greatly enhance a child’s learning journey and facilitate their integration into broader society.

### 6.5. Community and Educational Support

Schools and community programs play an essential role in reinforcing early intervention strategies. Well-trained educators can ensure that children with hearing loss are successfully integrated into classrooms, receiving the support they need to participate in both academic and social activities. Community-based programs, such as social skill development activities, also provide opportunities for children to practice learned behaviors in real-world settings, helping them gain confidence and develop essential social skills.

### 6.6. Ongoing Monitoring and Adaptive Assistance

Children’s needs evolve as they grow, and so must their intervention strategies. Regular, dynamic assessments are crucial to ensure that intervention plans remain relevant and effective as the child progresses through different developmental stages. If ongoing adjustments to hearing aids or cochlear implants are needed, they should be addressed promptly. By regularly evaluating a child’s progress, clinicians can make necessary adjustments to therapies and interventions, ensuring that the child receives the most appropriate, personalized support at every stage of their development.

## 7. Conclusions

Making NHS mandatory worldwide is both an ethical and social necessity. NHS enables early, timely interventions that empower children to develop essential language, cognitive, and social skills, setting them on a path toward full participation in society. Greece, with its ongoing NHS development, exemplifies both the challenges and the opportunities involved in establishing universal screening and could serve as a model for adapting NHS to diverse healthcare systems. Policymakers, healthcare providers, and international organizations must work together to make NHS a global standard, ensuring that every child—regardless of location or economic status—has access to this critical service. Universal NHS is not only a vital public health measure but also a fundamental step toward a more equitable and inclusive world, where every child is given the best possible start in life. No child should be left behind due to preventable hearing loss, and NHS is essential in realizing this vision.

## Figures and Tables

**Table 1:** Status of neonatal hearing screening (NHS) in Europe.

Countries	NHS Status
Albania	Under Development
Austria	Nationwide, Universal
Belgium	Nationwide, Universal
Bosnia and Herzegovina	Not nationwide, Universal
Bulgaria	Nationwide, Universal
Croatia	Nationwide, Universal
Cyprus	Nationwide, Universal
Czech Republic	Nationwide, Universal
Denmark	Nationwide, Universal
Estonia	Nationwide, Universal
Faroe Islands	Nationwide, Universal
Finland	Nationwide, Universal
France	Nationwide, Universal
Georgia	Nationwide, Universal
Germany	Nationwide, Universal
Greece	Under Development
Hungary	Nationwide, Universal
Iceland	Nationwide, Universal
Ireland	Nationwide, Universal
Italy	Not nationwide, Universal
Kosovo	No national NHS program, Services available privately
Latvia	Nationwide, Universal
Lithuania	Nationwide, Universal
Luxembourg	Nationwide, Universal
Malta	Not nationwide, Selective
Moldova	Not nationwide, Universal
Montenegro	Under Development
Netherlands	Nationwide, Universal
North Macedonia	Not nationwide, Selective
Norway	Nationwide, Universal
Poland	Nationwide, Universal
Portugal	Nationwide, Universal
Romania	Not nationwide, Universal
Russian Federation	Nationwide, Universal
Serbia	Not nationwide, Universal
Slovakia	Nationwide, Universal
Slovenia	Nationwide, Universal
Spain	Nationwide, Universal
Sweden	Nationwide, Universal
Switzerland	Nationwide, Universal
Turkey *	Nationwide, Universal
United Kingdom	Nationwide, Universal

* Turkey was included despite not being entirely within Europe geographically.

**Table 2:** Status of neonatal hearing screening (NHS) across continents.

Continents	NHS Status
**Africa**	
Algeria, Egypt, Kenya, Nigeria, South Africa, Uganda	Pilot screening programs
**North and South America**	
Argentina, Brazil, Chile, Mexico	Not nationwide, Universal
Canada	Not nationwide (varies by province), Universal
United States	Nationwide, Universal
**Asia**	
India, Thailand	Under Development
Japan, South Korea, China	Nationwide, Universal
**Australia and Oceania**	
Australia, New Zealand	Nationwide, Universal

Under Development: NHS is in the establishment or early implementation phase and is not yet widely or uniformly conducted nationwide. Nationwide, Universal: NHS is implemented across the entire country and includes all newborns, regardless of risk factors. Not nationwide, Universal: NHS is restricted to certain regions but includes all newborns within those areas. Not nationwide, Selective: NHS is regionally limited and focuses only on high-risk infants.

## Data Availability

No new data were created or analyzed in this study. Data sharing is not applicable to this paper.

## References

[1] Pimperton H., Blythe H., Kreppner J., Mahon M., Peacock J.L., Stevenson J., Terlektsi E., Worsfold S., Yuen H.M., Kennedy C.R. (2016). The impact of universal newborn hearing screening on long-term literacy outcomes: A prospective cohort study. Arch. Dis. Child..

[2] Morton C.C., Nance W.E. (2006). Newborn hearing screening-a silent revolution. N. Engl. J. Med..

[3] Association of Women’s Health, Obstetric and Neonatal Nurses (2022). Newborn screening. Nurs. Womens Health.

[4] Stone W.L., Basit H., Los E. (2024). Phenylketonuria. StatPearls.

[5] Bowden S.A., Goldis M. (2024). Congenital hypothyroidism. StatPearls.

[6] Harcke S.J., Rizzolo D., Harcke H.T. (2019). G6PD deficiency: An update. JAAPA.

[7] World Health Organization World Report on Hearing. https://www.who.int/publications/i/item/world-report-on-hearing.

[8] Boerrigter M.S., Vermeulen A.M., Benard M.R., van Dijk H.J.E., Marres H.A.M., Mylanus E.A.M., Langereis M.C. (2023). Cochlear implants or hearing aids: Speech perception, language, and executive function outcomes. Ear Hear..

[9] Parving A. (1999). The need for universal neonatal hearing screening-some aspects of epidemiology and identification. Acta Paediatr. Suppl..

[10] Korver A.M., Konings S., Dekker F.W., Beers M., Wever C.C., Frijns J.H., Oudesluys-Murphy A.M., DECIBEL Collaborative Study Group (2010). Newborn hearing screening vs. later hearing screening and developmental outcomes in children with permanent childhood hearing impairment. JAMA.

[11] Tomblin J.B., Harrison M., Ambrose S.E., Walker E.A., Oleson J.J., Moeller M.P. (2015). Language outcomes in young children with mild to severe hearing loss. Ear Hear..

[12] Yoshinaga-Itano C., Sedey A.L., Wiggin M., Mason C.A. (2018). Language outcomes improved through early hearing detection and earlier cochlear implantation. Otol. Neurotol..

[13] Vohr B., Jodoin-Krauzyk J., Tucker R., Topol D., Johnson M.J., Ahlgren M., Pierre L.S. (2011). Expressive vocabulary of children with hearing loss in the first 2 years of life: Impact of early intervention. J. Perinatol..

[14] Shukla A., Harper M., Pedersen E., Goman A., Suen J.J., Price C., Applebaum J., Hoyer M., Lin F.R., Reed N.S. (2020). Hearing loss, loneliness, and social isolation: A systematic review. Otolaryngol. Head Neck Surg..

[15] Young A., Gascon-Ramos M., Campbell M., Bamford J. (2009). The design and validation of a parent-report questionnaire for assessing the characteristics and quality of early intervention over time. J. Deaf Stud. Deaf Educ..

[16] Podury A., Jiam N.T., Kim M., Donnenfield J.I., Dhand A. (2023). Hearing and sociality: The implications of hearing loss on social life. Front. Neurosci..

[17] Idstad M., Engdahl B. (2019). Childhood sensorineural hearing loss and educational attainment in adulthood: Results from the HUNT study. Ear Hear..

[18] Kanji A., Khoza-Shangase K., Moroe N. (2018). Newborn hearing screening protocols and their outcomes: A systematic review. Int. J. Pediatr. Otorhinolaryngol..

[19] World Health Organization (2021). Hearing Screening: Considerations for Implementation.

[20] Wroblewska-Seniuk K.E., Dabrowski P., Szyfter W., Mazela J. (2017). Universal Newborn Hearing Screening: Methods and Results, Obstacles, and Benefits. Pediatr. Res..

[21] De Siati R.D., Rosenzweig F., Gersdorff G., Gregoire A., Rombaux P., Deggouj N. (2020). Auditory Neuropathy Spectrum Disorders: From Diagnosis to Treatment: Literature Review and Case Reports. J. Clin. Med..

[22] Akinpelu O.V., Peleva E., Funnell W.R., Daniel S.J. (2014). Otoacoustic Emissions in Newborn Hearing Screening: A Systematic Review of the Effects of Different Protocols on Test Outcomes. Int. J. Pediatr. Otorhinolaryngol..

[23] American Academy of Pediatrics, Joint Committee on Infant Hearing (2007). Year 2007 Position Statement: Principles and Guidelines for Early Hearing Detection and Intervention Programs. Pediatrics.

[24] Joint Committee on Infant Hearing (2019). Year 2019 Position Statement: Principles and Guidelines for Early Hearing Detection and Intervention Programs. J. Early Hear. Detect. Interv..

[25] Choe G., Park S.K., Kim B.J. (2023). Hearing Loss in Neonates and Infants. Clin. Exp. Pediatr..

[26] Vos B., Senterre C., Lagasse R., Tognola G., Levêque A. (2016). Organisation of Newborn Hearing Screening Programmes in the European Union: Widely Implemented, Differently Performed. Eur. J. Public Health.

[27] Neumann K., Mathmann P., Chadha S., Euler H.A., White K.R. (2022). Newborn Hearing Screening Benefits Children, but Global Disparities Persist. J. Clin. Med..

[28] Wen C., Zhao X., Li Y., Yu Y., Cheng X., Li X., Deng K., Yuan X., Huang L. (2022). A systematic review of newborn and childhood hearing screening around the world: Comparison and quality assessment of guidelines. BMC Pediatr..

[29] Peñaranda D., Vo R.H., Sih T., Gonzalez Franco G., Valdez T.A. (2024). Advancing Neonatal Hearing Screening in Latin America: Insights from Pediatric Otolaryngologists. Int. J. Pediatr. Otorhinolaryngol..

[30] Hollowell J.L., Takagi A. (2022). The Status of Newborn Hearing Screening in Japan: Past, Present, and the Future. Cureus.

[31] Wen C., Huang L.H. (2023). Newborn Hearing Screening Program in China: A Narrative Review of the Issues in Screening and Management. Front. Pediatr..

[32] Nikolopoulos T.P. (2015). Neonatal hearing screening: What we have achieved and what needs to be improved. Int. J. Pediatr. Otorhinolaryngol..

[33] EUsscreen Vision & Hearing Summary Hearing Screening Country Reports. https://www.euscreen.org/hearing-screening-country-reports.

[34] Kik J., Simonsz H.J. (2021). Report: EUSCREEN Final Dissemination Meeting in Zaandam on Monday, June 28th and Tuesday, 29th. Strabismus.

[35] Kik J., Heijnsdijk E.A., Mackey A.R., Carr G., Horwood A.M., Fronius M., Carlton J., Griffiths H.J., Uhlén I.M., Simonsz H.J. (2023). Availability of Data for Cost-Effectiveness Comparison of Child Vision and Hearing Screening Programmes. J. Med. Screen..

[36] Bussé A.M.L., Mackey A.R., Hoeve H.L.J., Goedegebure A., Carr G., Uhlén I.M., Simonsz H.J., EUSCREEN Foundation (2021). Assessment of Hearing Screening Programmes Across 47 Countries or Regions I: Provision of Newborn Hearing Screening. Int. J. Audiol..

[37] Mackey A.R., Bussé A.M.L., Hoeve H.L.J., Goedegebure A., Carr G., Simonsz H.J., Uhlén I.M., EUSCREEN Foundation (2021). Assessment of Hearing Screening Programmes Across 47 Countries or Regions II: Coverage, Referral, Follow-Up and Detection Rates from Newborn Hearing Screening. Int. J. Audiol..

[38] Sharma R., Gu Y., Ching T.Y.C., Marnane V., Parkinson B. (2019). Economic Evaluations of Childhood Hearing Loss Screening Programmes: A Systematic Review and Critique. Appl. Health Econ. Health Policy.

[39] Blanař V., Škvrňáková J., Pellant A., Vodička J., Praisler J., Boháčová E., Dršata J., Šenkeřík M., Chrobok V. (2021). Effectiveness of Neonatal Hearing Screening System: A 12-Year Single Centre Study in the Czech Republic. J. Pediatr. Nurs..

[40] EUsscreen Vision & Hearing. https://www.euscreen.org.

[41] Chadha S., Cieza A. (2018). World Health Organization and Its Initiative for Ear and Hearing Care. Otolaryngol. Clin. N. Am..

[42] Vernier L.S., Fernandes C.P., Skorin P.P., Ávila A.T.V., Levandowski D.C. (2024). Cost-effectiveness of Neonatal Hearing Screening Programs: Systematic Review. Int. Arch. Otorhinolaryngol..

[43] Muñoz K., Nelson L., Blaiser K., Price T., Twohig M. (2015). Improving Support for Parents of Children with Hearing Loss: Provider Training on Use of Targeted Communication Strategies. J. Am. Acad. Audiol..

[44] Renauld J.M., Basch M.L. (2021). Congenital Deafness and Recent Advances Towards Restoring Hearing Loss. Curr. Protoc..

[45] Xiong X., Xu K., Chen S., Xie L., Sun Y., Kong W. (2019). Advances in Cochlear Implantation for Hereditary Deafness Caused by Common Mutations in Deafness Genes. Chin. Med. Assoc..

[46] Shi X., Liu X., Sun Y. (2023). The Pathogenesis of Cytomegalovirus and Other Viruses Associated with Hearing Loss: Recent Updates. Viruses.

